# Oxidative Imbalance and Kidney Damage: New Study Perspectives from Animal Models to Hospitalized Patients

**DOI:** 10.3390/antiox8120594

**Published:** 2019-11-28

**Authors:** Daniela Pellegrino, Daniele La Russa, Alessandro Marrone

**Affiliations:** 1 Department of Biology, Ecology and Earth Sciences, University of Calabria, 87036 Rende, Italy; alessandro.marrone@unical.it; 2 Analysis and Research on Oxidative Stress Laboratory (LARSO), University of Calabria, 87036 Rende, Italy; daniele.larussa@unical.it; 3 Department of Pharmacy, Health and Nutritional Sciences, University of Calabria, 87036 Rende, Italy

**Keywords:** chronic kidney disease, redox balance, inflammation, kidney damage

## Abstract

Chronic kidney disease (CKD) is a major public health problem worldwide and affects both elderly and young subjects. Its main consequences include the loss of renal function, leading to end-stage renal disease, an increased risk of cardiovascular disease, a significant increase in morbidity and mortality, and a decrease in health-related quality of life. This review arose in significant part from work in the authors’ laboratory, complemented by literature data, and was based on a translational approach: we studied the role of many CKD risk factors, such as hypertension, obesity, and oxidative stress/inflammation. The aim was to identify new molecular mechanisms of kidney damage to prevent it through successful behavior modifications. For this purpose, in our studies, both human and animal models were used. In the animal models, we analyzed the mechanisms of renal damage induced by hypertension (spontaneously hypertensive rats) and obesity (cafeteria diet-fed rats), showing that redox disequilibrium in plasma and tissue is extremely important in renal alteration in terms of both oxidative damage (lipid peroxidation, altered expression antioxidant enzymes) and apoptotic pathway (intrinsic/extrinsic) activation. In hemodialysis patients, we explored the correlation between the global oxidative balance and both inflammatory markers and cardiovascular risk, showing a strong correlation between the oxidative index and the blood levels of C-reactive protein and previous cardiovascular events. This multilevel approach allowed us to individually and synergistically analyze some aspects of the complex pathogenic mechanisms of CKD in order to clarify the role of the new amplified risk factors for CKD and to prepare an effective personalized prevention plan by acting on both modifiable and nonmodifiable risk factors.

## 1. Introduction

Oxidative stress, a disturbance in the complex pro-/antioxidant balance, is widely recognized as a critical component of the pathogenesis and progression of chronic kidney disease (CKD) [1,2]. Due to its high metabolism, the kidney is extremely vulnerable to oxidative damage, and several experiments have shown that oxidative stress can cause/accelerate both disease progression and complications [1,3]. Despite several experimental and clinical studies having explored the intricate mechanisms between CKD and oxidative imbalance, the pathophysiological mechanisms of organ damage have not been clarified. This review aims to outline the current understanding on molecular mechanisms of oxidative kidney damage and to highlight the potential targets for therapeutic intervention.

## 2. Oxidative Stress and Antioxidant Defense Mechanisms

Oxidative stress occurs when there is an imbalance between the production of free radical species and the antioxidant ability to neutralize their harmful effects [4]. Free radicals can be defined as highly reactive molecular species (atoms or molecules) that contain one or more unpaired electrons in their external shell or outer orbit and that are capable of independent existence [5]. In cells, these radicals can act as oxidants or reductants by losing or accepting a single electron, and they are continuously produced by the organism’s normal use of oxygen [6]. Free radicals include reactive radical and nonradical derivatives of oxygen (ROS) and nitrogen (RNS) that are collectively called reactive oxygen nitrogen species (RONS) [7]. The generation of RONS is a physiological process and, at moderate or low levels, RONS are important molecules involved in a number of cellular signaling pathways, in the extraction of energy from organic molecules, in immune defense, in mitogenic response, and in redox regulation [8]. An excess production or a decreased scavenging of RONS has been implicated in aging and age-related diseases [9]. Both endogenous and exogenous sources of RONS have been described. The endogenous sources of RONS include different subcellular organelles, such as mitochondria, peroxisomes, and endoplasmic reticulum, where oxygen consumption is high [10]. NADPH oxidase (nicotinamide adenine dinucleotide phosphate oxidase) is a prevalent source of the superoxide radical (•O^2−^), which is formed by the addition of one electron leak from the electron transport system during cellular respiration to the molecular oxygen. Most of the superoxide dismutates into hydrogen peroxide (H_2_O_2_) through superoxide dismutase (SOD) [8]. H^2^O^2^ is a neutral molecule because it has no unpaired electrons, but it is able to form the most reactive and dangerous radical, the hydroxyl radical (•OH), through a Fenton or Haber–Weiss reaction. Hydroxyl radicals mainly react with phospholipids in cell membranes and proteins. In activated neutrophils, in the presence of chloride and myeloperoxidase, H^2^O^2^ can be converted into hypochlorous acid that can react with DNA and produce pyrimidine oxidation products and add chloride to DNA bases [11]. Another important determinant in the cellular redox equilibrium is nitric oxide (NO). In mammals, NO can be generated by three main isoforms of nitric oxide synthase (NOS): endothelial NOS, which is related to vasodilation and vascular regulation; neuronal NOS, which is linked to cellular signaling; and inducible NOS, which is activated in response to various endotoxin or cytokine signals [12]. All isoforms of NOS utilize arginine as the substrate and molecular oxygen and reduced nicotinamide–adenine–dinucleotide phosphate (NADPH) as cosubstrates. The reaction of NO with the superoxide radical (•O^2−^), forms the potent oxidant peroxynitrite (ONOO^−^). This compound can cause oxidative damage, nitration, and the S-nitrosylation of biomolecules, including proteins, lipids, and DNA [13]. Nitrosative stress through ONOO^−^ has been implicated in DNA single-strand breakage, followed by poly-ADP-ribose polymerase (PARP) activation [14]. Exogenous sources of RONS are numerous and include air and water pollution, pesticides, tobacco, alcohol, heavy metals (Fe, Cu, Co, and Cr) or transition metals (Cd, Hg, Pb, and As), drugs (cyclosporine, tacrolimus, gentamycin, and bleomycin), industrial solvents, cooking (smoked meat, waste oil, and fat), and radiation. Inside the body, all of these substances are metabolized into free radicals [10]. Endogenous or exogenous RONS are capable of damaging biologically relevant molecules with consequent cell damage and homeostatic disruption [15]. Among them, lipids, carbohydrates, nucleic acids, and proteins are the major targets, and their oxidative modification can also be used as markers of oxidative stress [16].

Free radicals can damage cells through several mechanisms:

(1) Lipid peroxidation and loss of membrane fluidity: double bonds in polyunsaturated membrane lipids are vulnerable to attacks by oxygen-free radicals;
(2) Protein cross-linking: free radicals promote sulfhydryl-mediated protein crosslinking, resulting in increased degradation or loss of activity;
(3) DNA fragmentation;
(4) Oxidative damage to carbohydrates impairs the functions of some cellular receptors, including those associated with hormonal and neurotransmitter responses.

**Figure 1:**
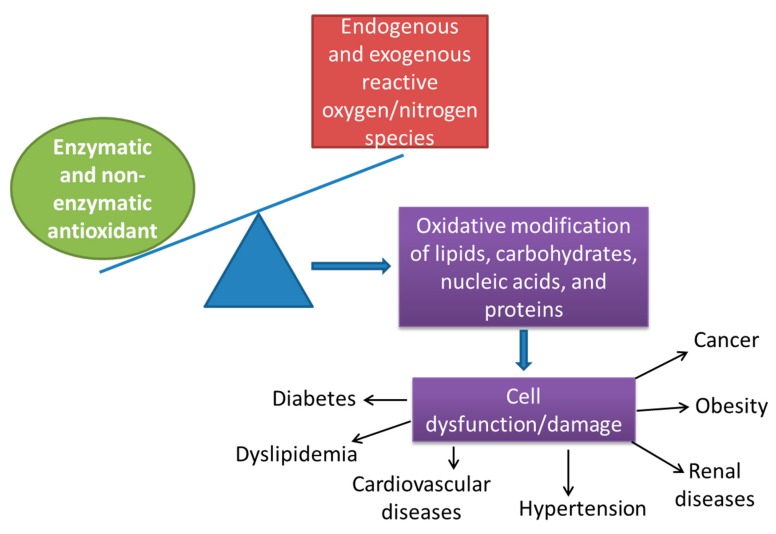
The imbalance between free radicals and antioxidant systems induces cell injury with consequent organ/system pathogenesis.

The overproduction of oxygen-derived free radicals has been implicated in the pathogenesis of over 200 clinical conditions (Figure 1). Tissue injury and its healing are characterized by a sequence of various events influenced by the cause of the injury and other factors, such as the intensity of the damaging agent, the type of tissue, and the condition of the whole organism. The healing process is mediated by a variety of messengers released by the immune system; for example, phagocytes produce cytotoxic agents, which not only prevent the spread of infection but also remove host cellular particles that are damaged [17]. The most important cellular defense mechanism is represented by antioxidant systems. The cells contain important antioxidant defense mechanisms that protect against free radical toxicity and include both endogenous and exogenous molecules. Endogenous antioxidants (naturally generated in situ) include enzymatic and nonenzymatic molecules. The primary enzymatic scavengers are superoxide dismutase (SOD), catalase (CAT), and glutathione peroxidase (GSH-Px). SOD catalyzes the dismutation of superoxide into hydrogen peroxide, which is decomposed into water and oxygen through CAT. In addition, GSH-Px converts peroxides and hydroxyl radicals into nontoxic forms through the oxidation of reduced glutathione (GSH) into glutathione disulfide, which is further reduced to GSH through glutathione reductase [18]. Nonenzymatic antioxidants are molecules such as glutathione, L-arginine, CoQ10, melatonin, albumin, and uric acid (85% of antioxidant capacity in plasma), which interact with RONS and terminate the free radical chain reactions [19]. Exogenous no-enzymatic antioxidants, which are supplied through foods, include ascorbic acid (vitamin C), which scavenges hydroxyl and superoxide radicals; α-tocopherol (vitamin E), which protects against the lipid peroxidation of cell membranes; phenolic antioxidants (resveratrol, phenolic acids, and flavonoids); lecithin oil; selenium; zinc; and drugs such as acetylcysteine [20].

## 3. Pathophysiology of Chronic Kidney Disease

CKD is recognized as a global health problem with a high rate of morbidity and mortality and elevated healthcare costs [21]. CKD affects 10–16% of the adult population around the world [22], with a mortality rate of 109.7 per 1000 patients/year [23]. A recent meta-analysis of observational studies reported that CKD has a high global prevalence (between 11% and 13%), with a higher percentage in developed areas such as Europe, the USA, Canada, and Australia, where the elderly population is greater than in developing areas [24]. Although many young patients are affected by CKD due to congenital disorders (glomerulonephritis and type I diabetes), the risk of CKD increases with age, and elderly patients are overrepresented in the dialysis population [25]. The main clinical manifestation of CKD is the loss of the glomerular filtration rate (GFR), which allows for the staging of CKD, with progressively decreasing (estimated) GFRs. According to the National Kidney Foundation, the Kidney Disease Outcomes Quality Initiative, and the Kidney Disease-Improving Global Outcomes convention, CKD is subdivided into five different stages: the first two stages have normal (GFR ≥ 90 mL/min/1.73 m^2^) or mild reduced kidney function (GFR between 60 and 89 mL/min/1.73 m^2^), while stages 3–5 have a severe reduction of kidney function (GFR 15–29 mL/min/1.73 m^2^), which leads to end-stage renal disease (ESRD).

**Figure 2:**
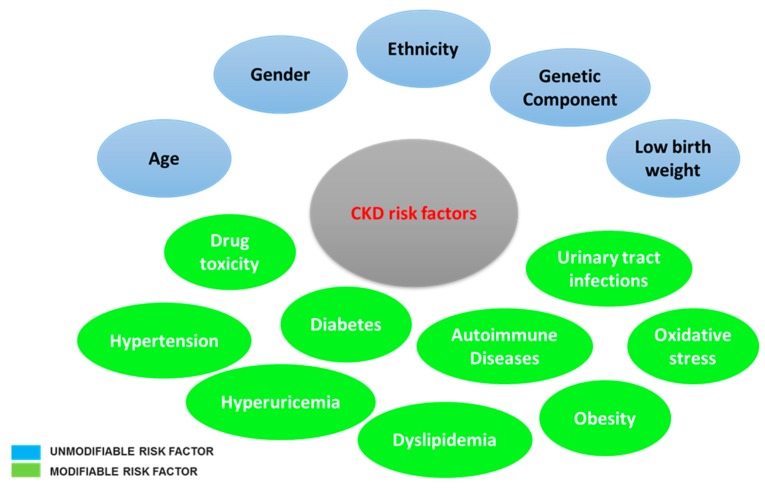
Chronic kidney disease (CKD) unmodifiable and modifiable risk factors.

The initiating causes of CKD are highly variable, since epidemiological studies have revealed that with CKD, unmodifiable and modifiable risk factors among patients can be defined (Figure 2). The first include age, gender, ethnicity, genetic components, and low birth weight; the second comprise drug toxicity, inflammation, obesity, oxidative stress, hyperuricemia, hypertension, dyslipidemia, autoimmune diseases, and urinary tract infections [26,27,28]. The pathophysiology of CKD involves two mechanisms: the initial mechanism of the specific underlying etiology (as immune complex glomerulonephritis or the exposure to toxins in some renal tubules (and interstitial disease)) and a series of progressive mechanisms involving the hyperfiltration and hypertrophy of the remaining viable nephrons. In addition, inflammation causes epithelial–mesenchymal transitions in renal tubular cells that move away from the basal membrane and form new interstitial fibroblasts that lead to tissue fibrosis. Interstitial fibrosis seems to drive further nephron injury through the promotion of renal ischemia [29]. Remaining viable nephrons lose the ability to perform autoregulation, resulting in systemic hypertension, which will ultimately be more damaging to the glomerulus and worsen CKD progression. There are many nonhemodynamic factors that play a role in CKD progression, such as angiotensin II, aldosterone, endothelin, acidosis, and oxidative stress. Angiotensin II contributes to the inflammation process by activating cytokines, adhesion molecules, transcription factors, and monocytes. Angiotensin II also increases the synthesis of the extracellular matrix, the hydraulic pressure of the glomerulus, and podocyte cell damage [30]. Aldosterone amplifies glomerular injury through the proliferation of mesangial cells, apoptosis, hypertrophy, and podocyte cell damage. Moreover, aldosterone causes structural and functional damage to blood vessels by acting as an angiotensin II mediator [30]. Endothelin is a potent vasoconstrictor whose levels increase during CKD, and it causes higher pressure in efferent blood vessels than in afferent blood vessels, thus resulting in increased glomerular hydraulic pressure [21]. Metabolic acidosis, which is due to a compromised capacity of the kidney to excrete ammonium or reabsorb bicarbonate, is a common complication of CKD, particularly in patients with a GFR below 20%. The increased ammonia production activates the alternative complement pathway, while the acidosis status stimulates the formation of both endothelin and aldosterone, which promote renal fibrosis [21].

## 4. Oxidative Stress and Chronic Kidney Disease

Over the last few years, several experimental studies have proven that oxidative imbalance is a common feature in renal diseases, representing a key process in the development and complications of CKD [3,31,32]. Oxidative stress could be both a potential cause and a consequence of renal function alteration, since the primary effect of proper redox regulation is to keep the balance of electrolytes and physiological buffer systems [33]. In addition, kidneys excrete toxins and waste metabolites that, if accumulated, could alter the redox homeostasis [34,35]. Among the putative mechanisms that contribute to the pathogenesis of CKD, oxidative stress has been recognized as accelerating disease progression through cardiovascular complications, inflammation, fibrosis, and apoptosis, as well as through glomerular filtration barrier damage [21,36]. For example, the glomerulosclerosis process, which is associated with an increase in oxidative stress, is induced by both increased transforming growth factor β (TGFβ) expression and reduced nitric oxide production/activity, causing tubulointerstitial fibrosis and inducing tubular destruction [37,38,39].

In particular, cardiovascular diseases are an important complication for CKD patients and represent the main causes of morbidity and mortality in these sick subjects [40,41]. Renal dysfunction represents an independent risk factor for cardiovascular disease in each CKD stage, as this population presents higher rates of diabetic, dyslipidemic, and hypertensive subjects [42,43,44].

In our laboratory, we focused on human studies regarding an evaluation of the overall redox/inflammatory state in a significant hemodialysis population, including subjects both with and without previous cardiovascular events [45]. Several authors have reported a profound imbalance between oxidants and antioxidants in CKD, though studies on plasmatic oxidative balance have shown conflicting results [36,46,47,48]. Moreover, it is unclear at which stage of renal deficiency the redox imbalance becomes more intense and if dialytic treatment increases redox imbalance [35,49,50]. Our results showed that the oxidative disequilibrium in hemodialysis patients was represented by an enhancement of the plasmatic antioxidant barrier effectiveness, which was significantly higher compared to the healthy controls. Although all hemodialysis patients possessed good plasmatic redox status, we detected a strong correlation between the oxidative index and C-reactive protein blood levels. This result was not surprising, since CKD is characterized by chronic inflammation [51] and oxidative stress is one of the key factors in triggering the inflammatory process [52,53]. In addition, this constant inflammatory status in hemodialysis patients is related to several comorbidities, mainly cardiovascular events [52]. The most interesting finding that emerged from our study is that, in our cohort, patients with previous cardiovascular diseases had higher values of both oxidative stress and antioxidant barriers with respect to subjects without cardiovascular events [45]. Our results suggest that the preservation of a redox balance can be considered a target for the prevention of cardiovascular complications during CKD progression.

**Figure 3:**
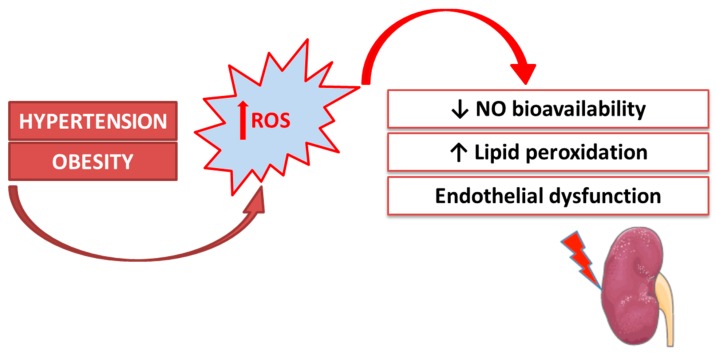
Oxidative stress is a crucial molecular mechanism in the pathogenesis of renal damage.

In animal models, our research focused on the correlation between oxidative imbalance and kidney damage by using experimental models of hypertension and obesity (Figure 3). Recent evidence has shown that oxidative stress is a crucial molecular mechanism involved in the pathogenesis of renal damage and that apoptosis occurs in critical organs (the heart, brain, and kidneys) during both hypertension and obesity [54,55]. The aim was to explore new molecular mechanisms of kidney damage to prevent it through successful behavior modifications. In both human and animal models, essential hypertension represents an important risk factor for renal dysfunction [56], though the correlation between elevated blood pressure and kidney damage has not been clarified [57,58]. Complex biochemical, hormonal, and hemodynamic mechanisms are involved in hypertensive organ damage [59], and a crucial role appears to be exerted by oxidative stress [60,61]. The pathophysiological role of oxidative imbalance has been reported in genetic and experimental models of hypertension and is linked to decreased NO bioavailability in the vasculature and kidneys [62]. In our study [54], we utilized a valid model of essential hypertension, the spontaneously hypertensive rat (SHR), at an age of 20 weeks, when hypertension is stably developed, vascular remodeling has started, but kidney function is preserved. In this experimental pathological model, we showed a significant alteration in both plasmatic pro-oxidant/antioxidant status and tissue oxidative damage. In particular, we detected a significant rise in lipid peroxidation levels in all SHR tissues, which was particularly relevant in the kidneys, and altered expression of the antioxidant enzymes superoxide dismutase 1 (SOD1) and glutathione S-tranferase P1 (GSTP1). In addition, in these hypertensive animals with preserved renal function, we found a strong activation of both intrinsic (liver and skeletal muscle) and extrinsic (kidney) apoptotic pathways [54]. These results suggest that, as well as having a direct effect on blood pressure, redox disequilibrium is extremely significant in hypertensive tissue alteration in terms of both oxidative damage and apoptotic pathway activation. Several studies have highlighted the solid correlation between obesity and chronic diseases, including cardiovascular disease [63,64], cancers [65,66,67], and renal diseases [68,69,70]. Obesity is a potent risk factor for kidney disease, as obese subjects have a greater chance of developing diseases such as diabetes and hypertension [68]. Several studies have also shown that being overweight or obese may directly cause renal dysfunction and kidney damage through a direct role in the endocrine activity of adipose tissue [71,72,73,74]. Indeed, many adipokines that are produced by higher visceral adipose tissue have been involved in the development of insulin resistance, inflammation, and oxidative stress [65,68,75,76]. In our experiments, we used an obesity model: cafeteria (CAF) diet-fed rats. In rodents, the CAF diet mimics diet-induced obesity in humans, inducing severe obesity, insulin resistance, and high plasma triglyceride levels [77,78,79]. To analyze obesity-induced organ damage, we hypothesized that the link between obesity and renal impairment could be represented by oxidative stress. The relationship between obesity and systemic oxidative stress has been described [80], and our results corroborated this assumption: the CAF diet induced a perturbation of the plasmatic oxidative equilibrium, with important antioxidant capacity depletion [55]. It is well known that increased oxidative stress in metabolic disorders (diabetes, obesity, and dyslipidemia) implies nonenzymatic antioxidant depletion [58], and this is the first detectable event of a redox disturbance (as has been highlighted by our research group in humans as well) [81,82]. In obesity models, the enzymatic antioxidant defenses turn out to be altered, but the scenario is very complex and dissimilar in different organs and tissues [83,84,85]. In renal tissues in our obesity model, we found no changes in the expression of SOD1, the most important preventive antioxidant, while there was a considerable and significant decrease in the GSTP1 monomer (the form with antioxidant and proliferative activity). These results support the recent genomic evidence that has highlighted the CAF diet-induced alterations in the white adipose gene transcriptome, which includes the important suppression of glutathione-related genes and pathways involved in mitigating oxidative stress [86]. In addition, in our model, we revealed the coexistence of oxidative imbalance and apoptosis activation, and this represents a potential mechanism of organ damage. The results from our group and others have suggested that the intricate link between obesity and kidney damage could be truly represented by a systemic oxidative imbalance, which is also underlined by the nephroprotective activity of substances with antioxidant activity [55,87]. In particular, we have shown that treatment with a bergamot polyphenolic fraction enhances the plasmatic ability to neutralize oxidative insults, mainly in the case of a redox disturbance due to the CAF diet [55].

## 5. Conclusions

CKD is a global health burden with a high economic cost to health systems, and its prevalence is expected to increase significantly in the coming years. It is necessary to carry out intervention strategies that are deliverable at scale to delay the onset/progression of CKD, and it is thus essential to have a deep understanding of all pathogenetic mechanisms. In obtaining this goal, basic and applied research and the interaction between them should be of equal importance The resulting synergy can help us to realize an effective personalized prevention plan by acting on both modifiable and nonmodifiable CKD risk factors.
